# Randomly Detected Genetically Modified (GM) Maize (*Zea mays* L.) near a Transport Route Revealed a Fragile 45S rDNA Phenotype

**DOI:** 10.1371/journal.pone.0074060

**Published:** 2013-09-09

**Authors:** Nomar Espinosa Waminal, Ki Hyun Ryu, Sun-Hee Choi, Hyun Hee Kim

**Affiliations:** 1 Plant Biotechnology Institute, Department of Life Science, Sahmyook University, Seoul, Korea; 2 Department of Plant Science, Plant Genomics and Breeding Institute and Research Institute for Agriculture and Life Sciences, College of Agriculture and Life Sciences, Seoul National University, Seoul, Korea; 3 Department of Horticulture, Biotechnology and Landscape Architecture, Seoul Women’s University, Seoul, Korea; Soonchunhyang University, Korea, Republic Of

## Abstract

Monitoring of genetically modified (GM) crops has been emphasized to prevent their potential effects on the environment and human health. Monitoring of the inadvertent dispersal of transgenic maize in several fields and transport routes in Korea was carried out by qualitative multiplex PCR, and molecular analyses were conducted to identify the events of the collected GM maize. Cytogenetic investigations through fluorescence *in situ* hybridization (FISH) of the GM maize were performed to check for possible changes in the 45S rDNA cluster because this cluster was reported to be sensitive to replication and transcription stress. Three GM maize kernels were collected from a transport route near Incheon port, Korea, and each was found to contain NK603, stacked MON863 x NK603, and stacked NK603 x MON810 inserts, respectively. Cytogenetic analysis of the GM maize containing the stacked NK603 x MON810 insert revealed two normal compact 5S rDNA signals, but the 45S rDNA showed a fragile phenotype, demonstrating a “beads-on-a-string” fragmentation pattern, which seems to be a consequence of genetic modification. Implications of the 45S rDNA cluster fragility in GM maize are also discussed.

## Introduction

Maize (*Zea mays* L.) is a critical source of animal feed, a staple food for many countries [1], and has many industrial uses such as production of adhesives, fuel, and sweeteners [2]. Maize also serves as a model species for studies of many basic biological processes [3]. More than a decade ago, the first genetically engineered maize was introduced to the market [4], and it has since been under continuous cultivation and selection. Major advantages of GM maize include agronomic traits such as weed control and resistance to pests. Based on the total area used for cultivation of biotech crops in 2012, insect resistant (Bt or IR) maize posted the sixth highest (4%), herbicide tolerant (HT) maize the fifth highest (5%), and stacked (Bt/HT, Bt/Bt/IR, and Bt/Bt/HT) the second (23%), only after soybeans (47%) [5]. Moreover, out of 159 Mha total global maize cultivation area, 55.1 Mha (35%) were utilized for GM maize cultivation in 2012 [5].

Crop genetic modification has been gaining popularity, but not without controversy. The main criticisms of this technique are focused on the environmental and health safety of GM crops carrying transgenes [6]. Major environmental concerns include the horizontal transfer of foreign genes such as HT genes to wild races and related species [7,8], the production of “super weeds” that are not killed by conventional doses of herbicides, and the effects of such crops on biodiversity via alterations in the food web [9–13]. Major health concerns include the potential for allergenicity and toxicity of new protein products [6].

In response to these concerns, the Cartagena Protocol on Biosafety was founded with the goal of controlling the transit and handling of living modified organisms (LMO) [14]. Several countries have required labeling products with GM ingredients, albeit with different thresholds of tolerance. For example, the European Union requires labels in products containing 0.9% GM ingredients, while Australia and New Zealand, Korea, and Japan require labels on products containing 1%, 3%, and 5% GM, respectively [15].

Korea, which is a major food-importing country [16], approved 54 GM crop events for food or feed consumption in 2010 [17], and this increased to 86 events in 2012, including 44 GM maize events [18]. Further increases may be approved in the near future as long as food self-sufficiency is not satisfied [16]. All approved GM crops were obtained from importation; however, the shipment and transit of these LMOs (in the form of seeds) could cause their inadvertent dispersal to the environment, raising the concern of an adventitious presence of genetically modified organisms (GMO) in the environment; hence, development of detection methods and monitoring of GM crops in Korea have been conducted [16,17,19].

Among various strategies employed for the detection and quantification of GMO, PCR-based methods are the most widely used [20]. Multiplex PCR uses several pairs of primers in one reaction to analyze a single template DNA and can simultaneously detect multiple target DNA segments in one tube [21]. The detection of GM maize using multiplex PCR has previously been conducted in Korea [22], and this technique has been used to monitor the presence of LMO that were inadvertently released into the environment [16,23]. In addition, Lee et al. [16] and Park et al. [23] recommended continuous monitoring for the presence of GM crops in Korea to regulate their unwanted spread in the environment.

A fragile site is a non-random, heritable [24] cytogenetic aberration commonly detected in mitosis [25]. In humans, a fragile site phenotype is said to be expressed when metaphase chromosomes exhibit gaps, constrictions, breaks or lesions, often in large regions of the genome [26]. Changes in the underlying DNA sequence and epigenetic modifications are associated with the expression of fragile sites [27,28]. Breakages at these sites have been linked with some genetic disorders and chromosome rearrangements in cancer cells [25,29,30]. In plants, very limited reports are available about chromosomal fragile sites, and their consequences in plant physiology are largely unknown [31].

The 45S rDNA cluster consists of repeat units of up to several thousands of copies in eukaryotic cells, and is known to be epigenetically controlled [32–36]. A few studies have reported observations of fragile sites in plants at the 45S rDNA loci, and these resembled those observed in humans [31,34,37]. Fragile 45S rDNA is characterized by chromosome lesions at this locus. This phenotype could occur naturally as in *Lolium* spp. However, fragile 45S rDNA differ in appearance from the secondary constriction of the nucleolar organizer region (NOR) such that they show not only the typical constrictions but also gaps or breaks connected by very thin chromatin fibers or none at all. This phenotype consequently resembles a bead-on-a-string morphology of the 45S rDNA locus at metaphase [33], and could also be observed in interphase nuclei [33,34,37].

But still, very little is known about the biological causes of 45S rDNA fragility. However, a recent study on barley (*Hordeum vulgare* L.), maize (*Zea mays* L.), rice (*Oryza sativa* L.), ryegrass ( *Lolium* *perenne* L.), and sorghum ( *Sorghum* *bicolor* L.) have shown that chemically induced replication and transcription stress caused epigenetic alterations leading to instability of the 45S rDNA cluster; thus, linking fragile site phenotype expression of the 45S rDNA locus to transcriptional defects and epigenetic alterations [33]. Furthermore, plant transformation is often associated with complex genome rearrangements and epigenetic alterations [38–40]. Taking these results in the context of genetic engineering, whether or not epigenetic changes caused by genetic modification could affect cytogenetic features of the 45S rDNA cluster is an intriguing subject for investigation.

In this study, we attempted to monitor the environmental dispersal of GM maize by multiplex PCR with maize samples collected from several sites in Korea, and conducted further molecular analyses to identify GM maize events. In addition, we carried out cytogenetic analyses through fluorescence in situ hybridization (FISH) to investigate the 45S rDNA cluster conditions in GM and non-GM maize samples.

## Materials and Methods

### Ethics statement

Field sampling in private farms were under the consent of farm owners. Other samples collected from public areas such as roads did not require specific permission. All samples were collected in non-protected areas and no endangered or protected species were involved.

### Sample collection

Maize samples were randomly collected from fields or roads in 14 different sites in Korea from early spring until late fall of 2010 (Table 1). In addition, seeds of a non-GM Korean maize cultivar ‘Mibaekchal’ were obtained from a farm in Kangwŏn Province, and seeds of cultivar ‘Paksachal’ were purchased from a seed shop. GM event MON810 was additionally collected in June, 2013, and its non-GM near isogenic line, Hi-II, was provided by PVGB, Seoul Women’s University. 'Mibaekchal' and GM sample 3 kernels were germinated in pots in the greenhouse, and crossed to obtain the F1 progeny. Root samples were obtained from pot-grown plants or petri dish-germinated seedlings.

**Table 1:** Summary of collected maize samples for GMO monitoring.

**Sampling location**	**(City)**	**Collection sites**	**Sites**
Gyeongsang Province	(Busan)	1	field
Kangwŏn Province	(Wonju)	5	field
Kyeonggi Province	(Yeoju)	1	field
	(Namyangju)	1	field
	(Incheon)	6	field or road
Total		14	

### Genomic DNA extraction and multiplex PCR

Leaves were collected directly from the sampling fields or obtained after germinating collected kernels and then frozen with liquid nitrogen and ground to a powder using a mortar and pestle. About 20-80 mg of powdered tissue were used for genomic DNA extraction according to the manufacturer’s protocol (Solgent, Korea, # SGD41-C100).

**Figure 1:**
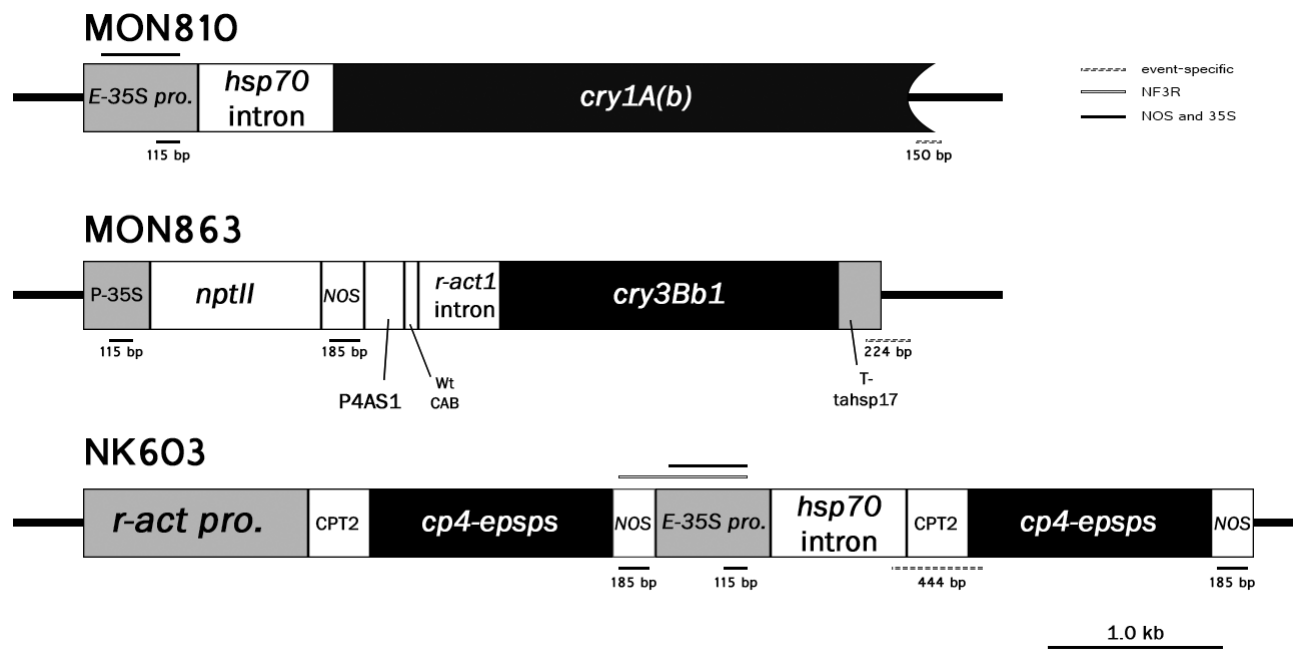
Diagram showing the GM-specific target amplification for each GM event. Expected amplicons for each primer set are shown below the construct diagram, while the amplicons for the enhanced 35S promoter and the NF3R are displayed above.

Two-step-down multiplex PCR (2SD-mPCR) was designed to optimally amplify target segments of transgenes using primers with different melting temperatures. The construct-specific primers used were specific for the *35S* promoter and *nos* terminator. Events MON810 and stacked MON863 x MON810 were previously detected in Incheon, Korea [16], so we used additional event-specific primers for MON810 and MON863 Shrestha et al. [41] (Figure 1 and Table S1). The maize constitutive *zein* gene was used as a positive control. Reactions were conducted in 25-µl mixtures consisting of 12.5 µl 2x Multiplex PCR Master Mix (Qiagen, USA, #206143), 10.75 µl DNase- and RNase-free water (Sigma, USA, #W4502), 1.25 µl 10x primer mix (Table S1) containing 0.2 µM of each primer, and ~50 ng of genomic DNA. The cycling conditions are presented in Table S2.

For singleplex PCR, one primer pair specific for the target sequence shown in Table S1 was used, while for quadruplex PCR, all except one of the five primer pairs were used. The PCR cycling conditions were the same as for 2SD-mPCR. PCR products were gel-analyzed using either 1% (w/v) SeaKem^®^ LE Agarose (Lonza, USA, #50004) or 2.5% (w/v) Metaphor^TM^ Agarose (Lonza, USA, #50180).

### NF3R sequencing and BLAST

After gel purification using a Wizard® SV Gel and PCR Clean-Up System (Promega, USA, #A9281), an aliquot of the NF3R amplicon was cloned into the pGEM®-T Easy Vector System II (Promega, USA, #A1380). Colonies that showed positive insertion after PCR were sent to Bionics Co., Ltd. (Korea) for sequencing, while another aliquot of the PCR products was sent for direct PCR sequencing and comparison. The consensus sequence was obtained from both cloned and directly sequenced amplicons. A BLASTn search for homology of the NF3R fragment was carried out using CLC Main Workbench against the nucleotide collection (nr) database at NCBI (http://www.ncbi.nlm.nih.gov/).

### Slide preparation and fluorescence in situ hybridization (FISH)

Healthy roots were collected and pretreated with 2 mM 8-hydroxyquinoline for 5 hours at 18°C, fixed in 90% acetic acid for 15 min at room temperature (RT, ~24°C), and then stored in 70% ethanol until use. Slide preparation was carried out according to the methods described by Waminal et al. [42–44].

For FISH, the slides were treated with 100 µg/ml RNase A (Sigma, USA, #R4875) in 2x SSC at 37°C for 1 hour, incubated in 4% paraformaldehyde in 2x SSC, dehydrated in ethanol series (70%, 90%, 100%), and air-dried. The hybridization mixture contained 50% formamide, 10% dextran sulfate, 2x SSC, 5 ng/µl salmon sperm DNA and 25 ng/µl of each probe DNA (biotin-labeled 45S rDNA and dig-labeled 5S rDNA) diluted to a total volume of 40 µl per slide with sterile water. The mixture was denatured at 90°C for 10 min and then kept on ice for at least 5 min. The chromosomes were subsequently denatured at 80°C for 5 min, after which slides were incubated in a humid chamber at 37°C overnight, and finally washed in 2x SSC at room temperature for 5 min and 0.1x SSC at 42°C for 35 min. For probe detection, biotinylated 45S rDNA was detected with streptavidin-Cy3 (Invitrogen, USA, SA1010), while digoxigenin-labeled 5S rDNA was detected with anti-dig-FITC (Sigma, USA, #F3523). Slides were then washed in TNT buffer (100mM Tris-HCl, pH 9.5; 100mM NaCl, and 0.2% Tween 20) at 37°C three times, after which they were subjected to an ethanol dehydration series (70%, 90%, 100%) and air dried. Finally, chromosomes were counterstained with premixed DAPI at 1 µg/ml (Roche, Germany, #10236276001) in Vectashield (Vector Laboratories, USA, # H-1000).

Chromosome spreads were examined and selected under an Olympus BX51 fluorescence microscope equipped with a CCD camera (CoolSNAP™ cf). Images were analyzed using Genus version 3.1 (Applied Imaging), and final images were edited using Adobe Photoshop CS6.

## Results

### Monitoring and detection of imported GM maize in transport routes and fields in Korea

**Figure 2:**
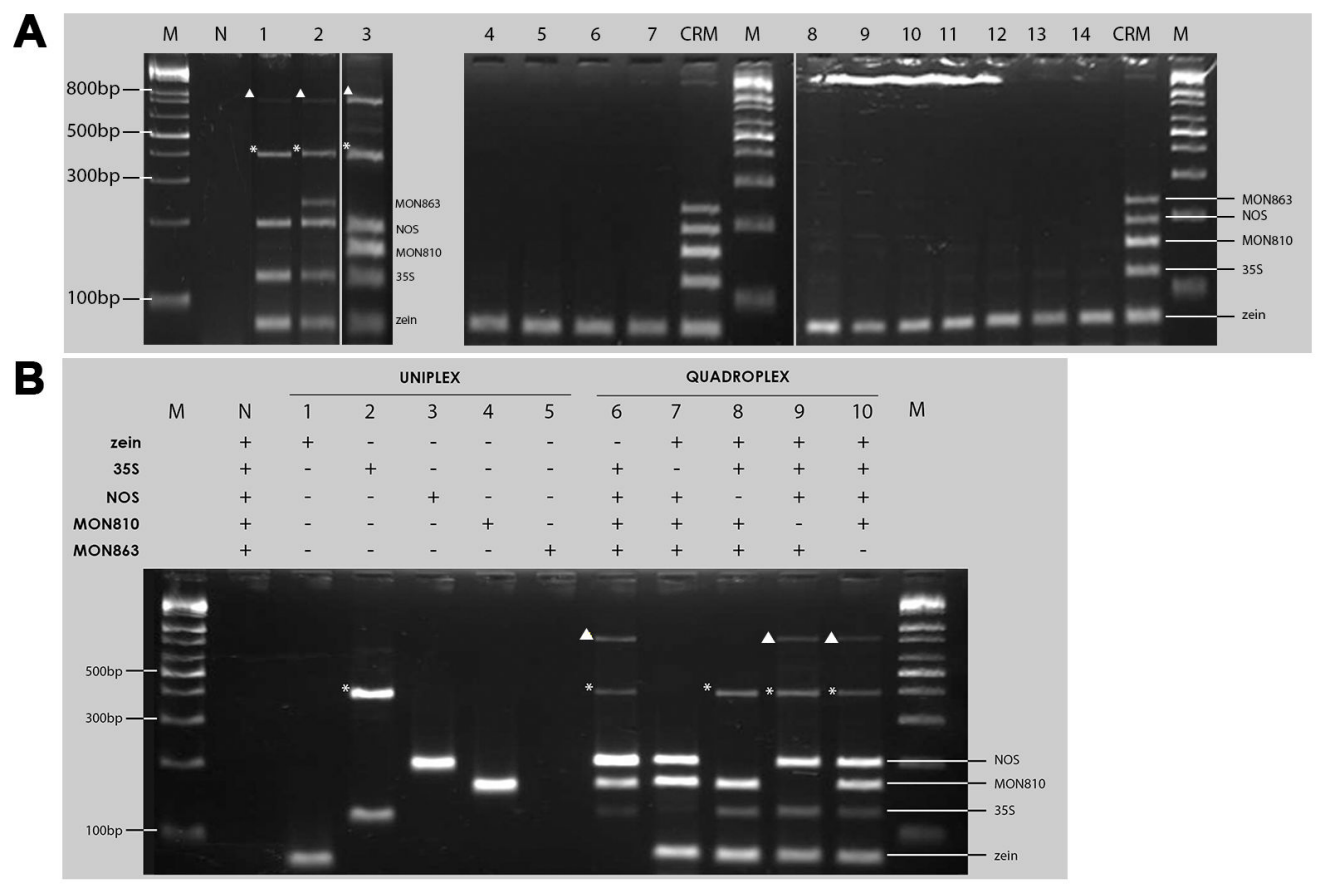
PCR analysis of the monitoring samples. Panel A reveals three GM events (lanes 1-3) based on amplification of the 35S promoter- and nos-specific bands. Asterisks and triangles indicate unexpected ~400 bp and ~800 bp bands, respectively. Lane N: negative control (no DNA template), M: 1kb+ DNA ladder, CRM: certified reference material with MON810 and MON863 DNA (Sigma Cat. No. ERMBF417D), 1-3: Yeonan Wharf road samples, 4-7 and 11: Wonju samples, 8-10: Incheon field samples, 12: Yeoju sample, 13: Namyangju sample, and 14: Busan sample. Zein was used as a positive control for maize. B: Comparison of the singleplex and quadruplex PCR results of GM sample 3. The absence of the ~800 bp band in reactions 7 and 8 indicates that the 35S and NOS primers are responsible for its amplification. Asterisks and triangles indicate unexpected ~400 bp and ~800 bp bands, respectively.

At one sampling site around the Yeonan Wharf in Incheon, many kernels were observed dispersed along the gutter of the road around a warehouse and on the soil adjacent to a road, although no established maize plants were observed in the vicinity. We confirmed three GM maize samples from this area, but no other field samples showed any transgene-specific bands (Figure 2A). All three detected GM samples showed bands for the *35S* promoter and the *nos* terminator; however, the presence of the MON810 and MON863 bands varied in these samples, indicating that they were distinct events. In Figure 2A, no bands corresponding to MON810 or MON863 were observed in lane 1 (GM sample 1), while only the MON863-specific band appeared in lane 2 (GM sample 2), and only the MON810 band was present in lane 3 (GM sample 3). Additionally, two non-target bands of about 400 bp and 800 bp (Figure 2A) were amplified from the three GM samples, suggesting that these bands could be caused by two outside primers amplifying closely adjacent target fragments. Considering these additional bands and the event-specific bands, these findings indicate that each sample is a unique event.

**Figure 3:**
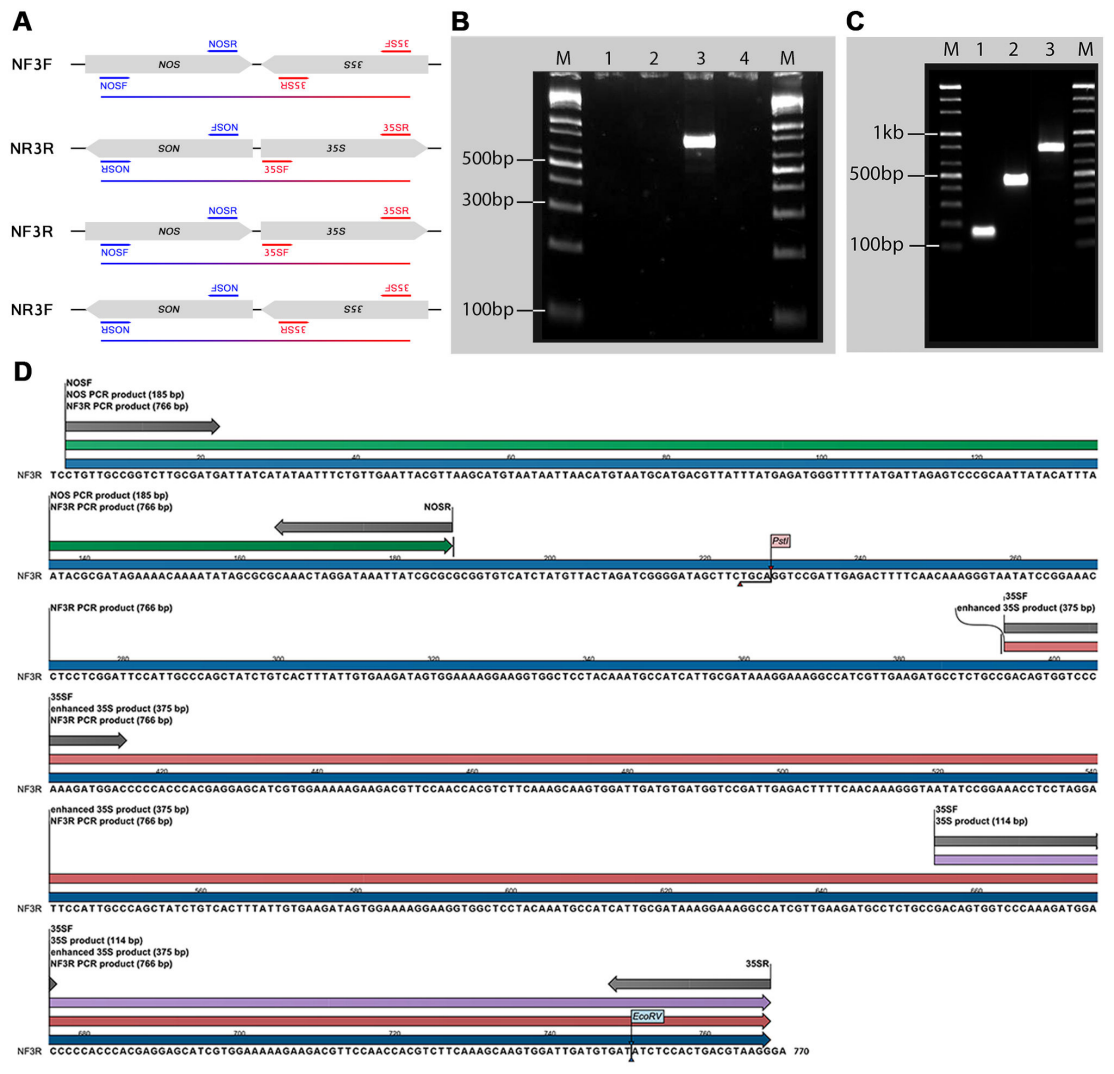
PCR and sequence analyses of the ~800 bp band. Panel A: Amplification diagram of the different combinations of 35S and NOS primers to investigate the primer pair responsible for amplification of the ~800 band and their physical orientation. B: PCR results of primer combinations from panel A. Lane 1: NOSF and 35SF (NF3F), 2: NOSR and 35SR (NR3R), 3: NOSF and 35SR (NF3R), and 4: NOSR and 35SF (NR3F). Only the NF3R combination produced the band, indicating the upstream location of the nos segment to the 35S. C: Confirmation of the NK603 event-specific cassette in GM sample 3. Among the different GM maize events, NK603 contains adjacent nos and 35S loci. The PCR results showed that the NF3R band corresponds to the NK603 event. Lane 1: MON810, 2: NK603-specific, 3: NF3R. These results also demonstrate the stacked event of GM sample 3. D: The consensus sequence of the NF3R band (766 bp, blue arrow). The sequence further confirmed the upstream location of the nos terminator (green arrow) to the 35S (114 bp, purple arrow) and *e35S* (375 bp, red arrow) promoters. The 35S and NOS primer pairs are shown as gray arrows.

To identify which outside primers in the cocktail caused the amplification of high-molecular-weight bands, we designed a comparative singlepex and quadruplex PCR using GM sample 3 as the template to also use the MON810 band as a control. Expected bands were observed in the singleplex PCR except for the additional ~400 bp in the 35S primer set reaction (Figure 2B). These findings indicate that one of the 35S primers has two possible binding sites, one amplifying the expected 115 bp fragment and the other the ~400 bp fragment. In the quadruplex PCR, all reaction mixtures with the 35S primer set showed the *35S* promoter-specific and the ~400 bp bands; however, the *nos* fragment was not amplified in the two reactions that did not contain the 35S and NOS primers. These results suggest that the three GM samples have inserts bearing closely adjacent loci for the *35S* promoter and *nos* terminator, and that one of the outside primers of these targets amplified the ~800-bp band. To simultaneously confirm the exact outside primers and the orientation of the amplified segments, we conducted singleplex PCR using the following primer sets: NOSF-35SF (NF3F), NOSR-35SR (NR3R), NOSF-35SR (NF3R), and NOSR-35SF (NR3F) (Figure 3A). Among the four pairs, only the NF3R primer set efficiently amplified the ~800-bp band (Figure 3B), indicating that the *nos* terminator is upstream of the *35S* promoter.

Sequencing of the NF3R band physically confirmed the sequence and distance between the *35S* promoter and *nos* segments, and the presence of an *enhanced 35S* promoter (*e35S*). The actual physical length of the NF3R band was confirmed to be 766 bp, which included the fragments specific to *nos* (185 bp), *35S* (114 bp), and the *e35S* (375 bp). The *nos* and *e35S* fragments were separated by 206 bp (Figure 3D).

### Identification of the events of each GM sample

A homology search of the NF3R fragment revealed that it matched expression vector pMON108080 (JN400381), covering 770 nt in region 8850-9620, showing an adjacent *nos* and *e35S* loci. Further review of published articles that present the structures of transgenic cassettes in commonly imported GM maize events [21,45,46] and the GM Crop Database [47] for identification of the adjacently localized *nos* terminator and *e35S* promoter resulted in detection of event NK603. This event has a 6706 bp transgene cassette containing two adjacent copies of genes encoding the 5-enolpyruvylshikimate-3-phosphate synthase (EPSPS) derived from *Agrobacterium tumefaciens* strain CP4 (CP4-EPSPS) and their corresponding expression regulators (Figure 1). The first CP4-EPSPS copy is terminated by the *nos* terminator, which is followed immediately by the *e35S* promoter for the second copy of the CP4-EPSPS.

To confirm the NK603 event, we performed PCR analysis of GM sample 3 using primers for NF3R and a new set of primers for NK603 designed by Onishi et al. [21] in separate PCR reactions. A band corresponding to NK603 was amplified along with the NF3R band (Figure 3C), indicating that the NF3R primer was not only useful for identification of the events among the unknown GM samples collected in Incheon, but also had potential utility for detection of event NK603.

These data suggest that the three GM samples we detected could be: GM sample 1: a single trait NK603 or other stacked GM maize events with the NK603 transgene locus, but not MON810 and MON863 (i.e. TC1507 x NK603, 59122 x NK603, MON89034 x NK603, MON89034 x TC1507 x NK603, NK603 x T25, or TC1507 x 59122 x NK603), GM sample 2: stacked event MON863 x NK603, or GM sample 3: either stacked NK603 x MON810 or multiple stacked events containing both MON810 and NK603, but not MON863 (i.e. TC1507 x MON810 x NK603, TC1507 x 59122 x MON810 x NK603, or TC1507 x 59122 x MON810 x MIR604 x NK603). All three samples were among the GM maize events approved for consumption in Korea [17,18].

### Cytogenetic investigation of 45S rDNA fragility

**Figure 4:**
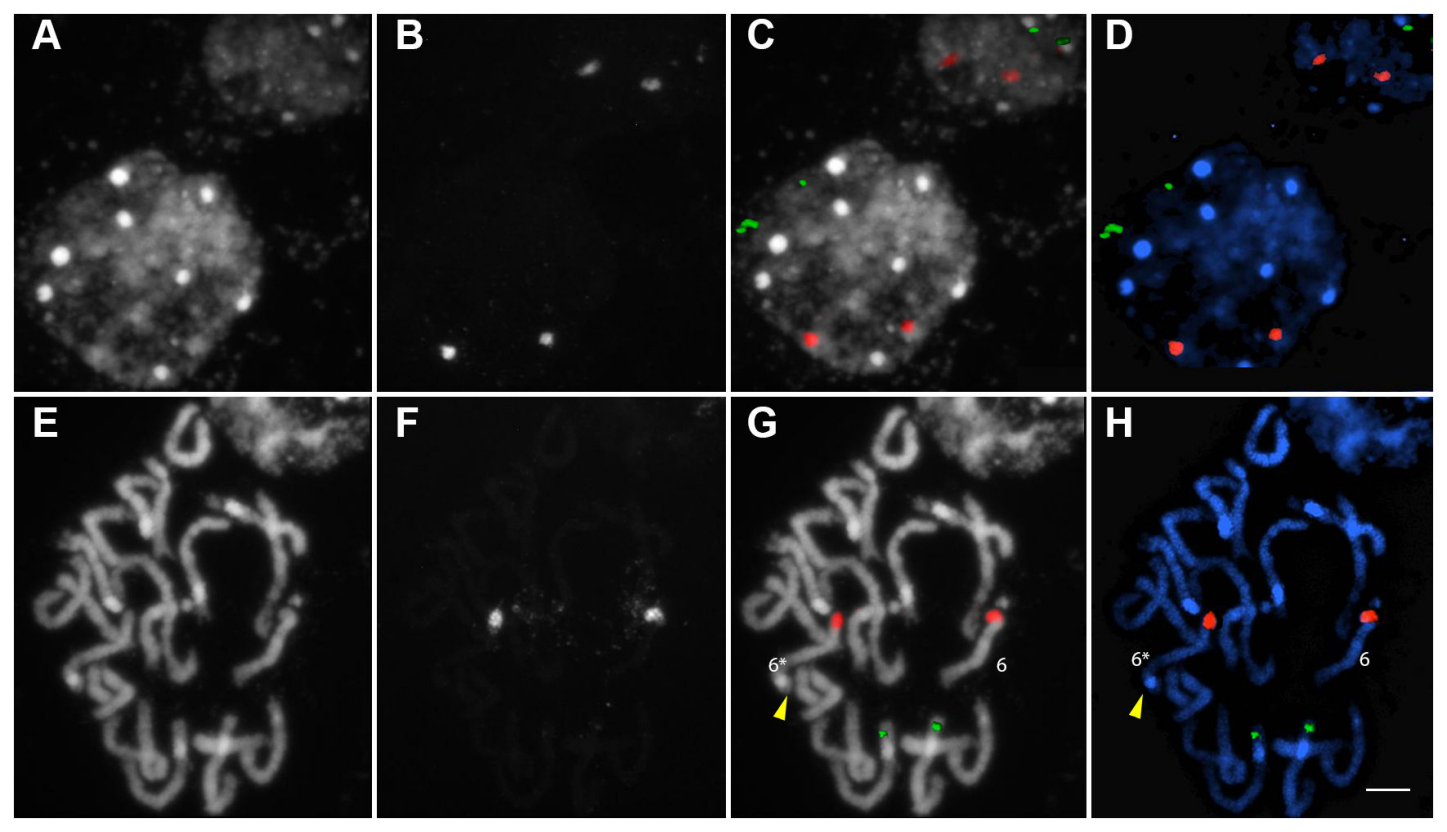
FISH analysis of the 45S rDNA cluster in ‘Mibaekchal’. The 5S (green) and 45S (red) rDNA probes showed two signals in both the interphase (A–D) and pro-metaphase chromosomes (E–H). Panels A and E: raw DAPI image, B and F: raw 45S rDNA signal, C and G: overlaid pseudo-colored rDNA signals on raw DAPI images, D and H: merged pseudo-colored images. Yellow arrowheads indicate the knob of a satellite homologue 6. Bar = 5µm.

**Figure 5:**
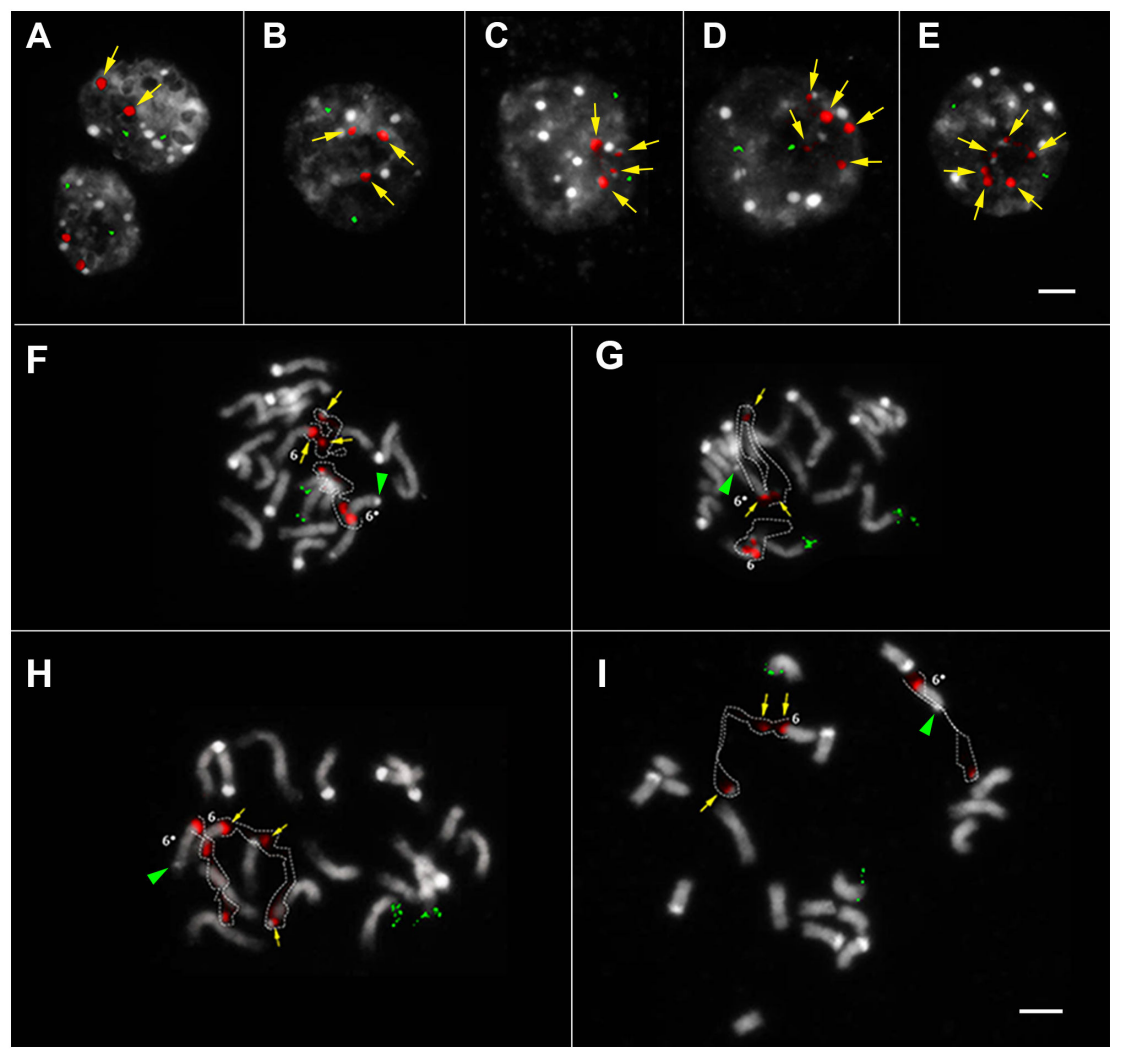
FISH analysis of the 45S rDNA cluster in GM sample 3. In both the interphase (A–E) and metaphase (F–I) spreads, two signals for the 5S rDNA cluster (green) but fragmented patterns for the 45S rDNA cluster (red) were observed. Panel A shows the normal two 45S rDNA signal pattern, while panels B-E show the different numbers of 45S rDNA fragments ranging from three to six. Yellow arrows emphasize the 45S rDNA signals, bar = 5µm. Panels F-I show different metaphase spreads displaying homozygously fragmented 45S rDNA with “beads” (yellow arrows) and “string” (broken lines) patterns. Satellite chromosomes are indicated by the number 6, and asterisks represent the homologue bearing a large knob (green arrowhead), bar = 5µm.

Further cytogenetic investigations were carried out for GM sample 3. The 5S rDNA locus number and condensation patterns were observed to be invariant in both ‘Mibaekchal’ and GM sample 3 (Figures 4 and 5). However, although the 45S rDNA locus was intact in ‘Mibaekchal’, a fragmented “beads-on-a-string” pattern was observed in GM sample 3 in both the interphase and metaphase spreads (Figures 4 and 5). In intact 45S rDNA, FISH signals appeared as big, bright, and compacted spots, compared with fragmented loci which were mostly smaller and weaker (Figure S1).

For the interphase cells examined at random, 207 (95%) out of 218 showed two distinct intact 45S rDNA signals for ‘Mibaekchal’ (Figure 4A–D, Table 2), while only six (5%) out of 117 were observed for the GM sample (Figure 5A, Table 2). Conversely, in the GM sample, 111 (95%) showed fragmented signals with a “beads-on-a-string” pattern ranging from two, with loose signals, to six fragments (Figure 5B–E, Table 2). Among the fragmented 45S rDNA in interphase nuclei, those consisting of four fragments were most frequently observed (29%) (Figure 5 and Figure S1, Table 2).

**Table 2:** Summary of 45S rDNA cluster fragility on interphase and metaphase spreads between 'Mibaekchal', GM Sample 3, and their F_1_ progeny.

**Sample**	**Cell phase**	**Intact^a^**	**Fragmented**		**Total**
‘Mibaekchal’	interphase	207 (95.0)^b^	11 (5.0)		218
	metaphase	53 (93.0)	4 (7.0)		57
	subtotal	260 (94.5)	15 (5.5)		275
GM Sample 3	interphase	6 (5.1)	111 (94.9)		117
			U^c^	41	
			3	26	
			4	29	
			5	14	
			6	1	
	metaphase	1 (1.9)	53 (98.1)		54
	subtotal	7 (4.1)	164 (95.9)		171
F_1_	interphase	8 (3.3)	233 (96.7)^d^		241
	metaphase	3 (4.7)	61 (95.3)		64
	subtotal	11 (3.6)	294 (96.4)		305
Total					751

a Two distinct signals

b Values inside the parentheses are percentages

c Uncategorized: number of loci are either two with many small dispersed fragments or totally dispersed signals

d Three signals

**Figure 6:**
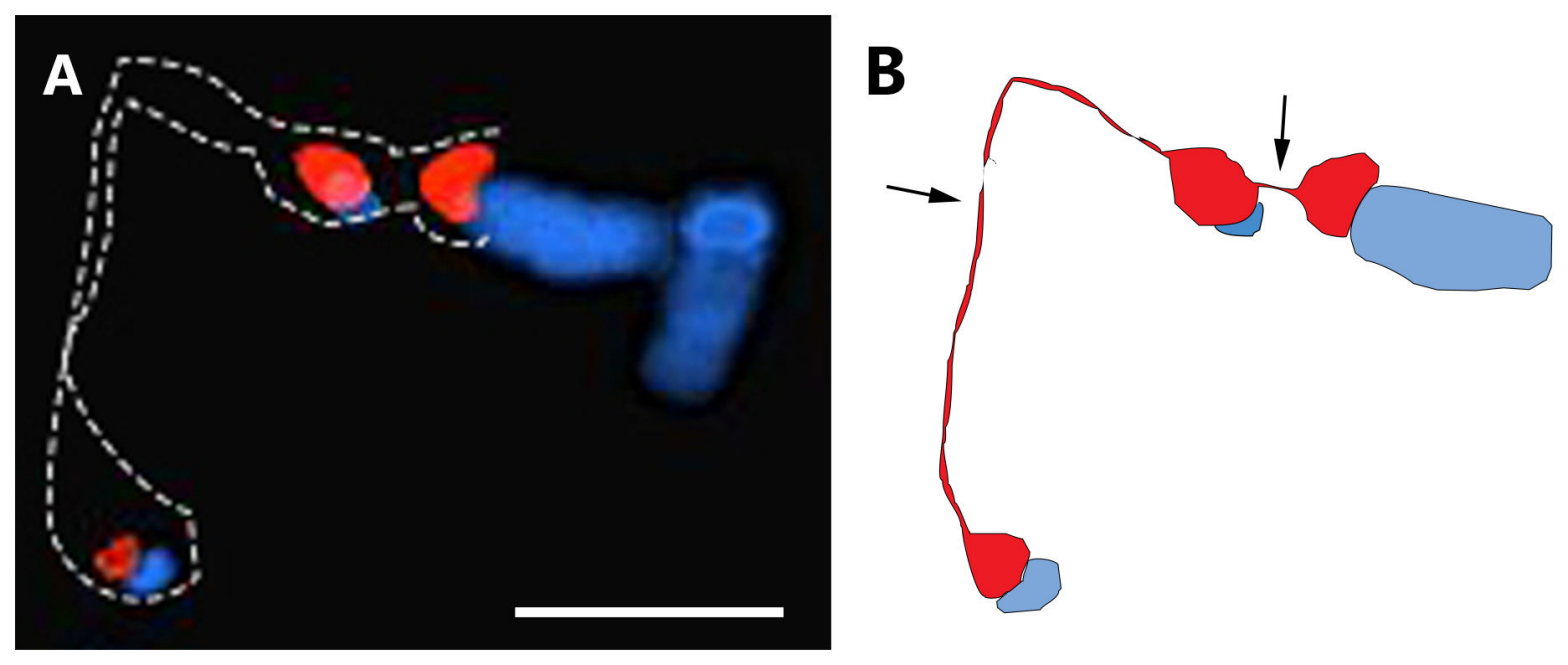
Diagram showing the “beads-on-a-string” pattern observed in GM sample 3. The 45S rDNA cluster in the GM sample frequently revealed three “beads” connected by two “strings” (arrows) in metaphase spreads. Panel A: part of a metaphase spread (full image shown in Figure S2, panel D), B: diagram of the image in panel A. Bar = 5µm.

For the metaphase cells, 53 (93%) out of the 57 spreads showed two intact signals for 45S rDNA, while only four (7%) showed signal fragmentation for ‘Mibaekchal’ (Table 2). However, for the GM sample, 45S rDNA fragmentation was frequently observed, in many cases forming three signal “beads” connected by two loose chromatin “strings” (Figures 5, 6, Figure S2, and Figure S3). Among 54 metaphase spreads observed, 53 (98%) revealed fragmented 45S rDNA. Indeed, even the condensed metaphase chromosome spread used for karyotyping showed loose rDNA chromatin with the frequently observed “beads-on-a-string” pattern, indicating non-synchronized/stalled and unequal condensation of the rDNA cluster (Figure S3 and Figure S4). The karyotypes of ‘Mibaekchal’ and the GM sample are presented in Figure S4.

### Heritability of the fragile 45S rDNA phenotype

**Figure 7:**
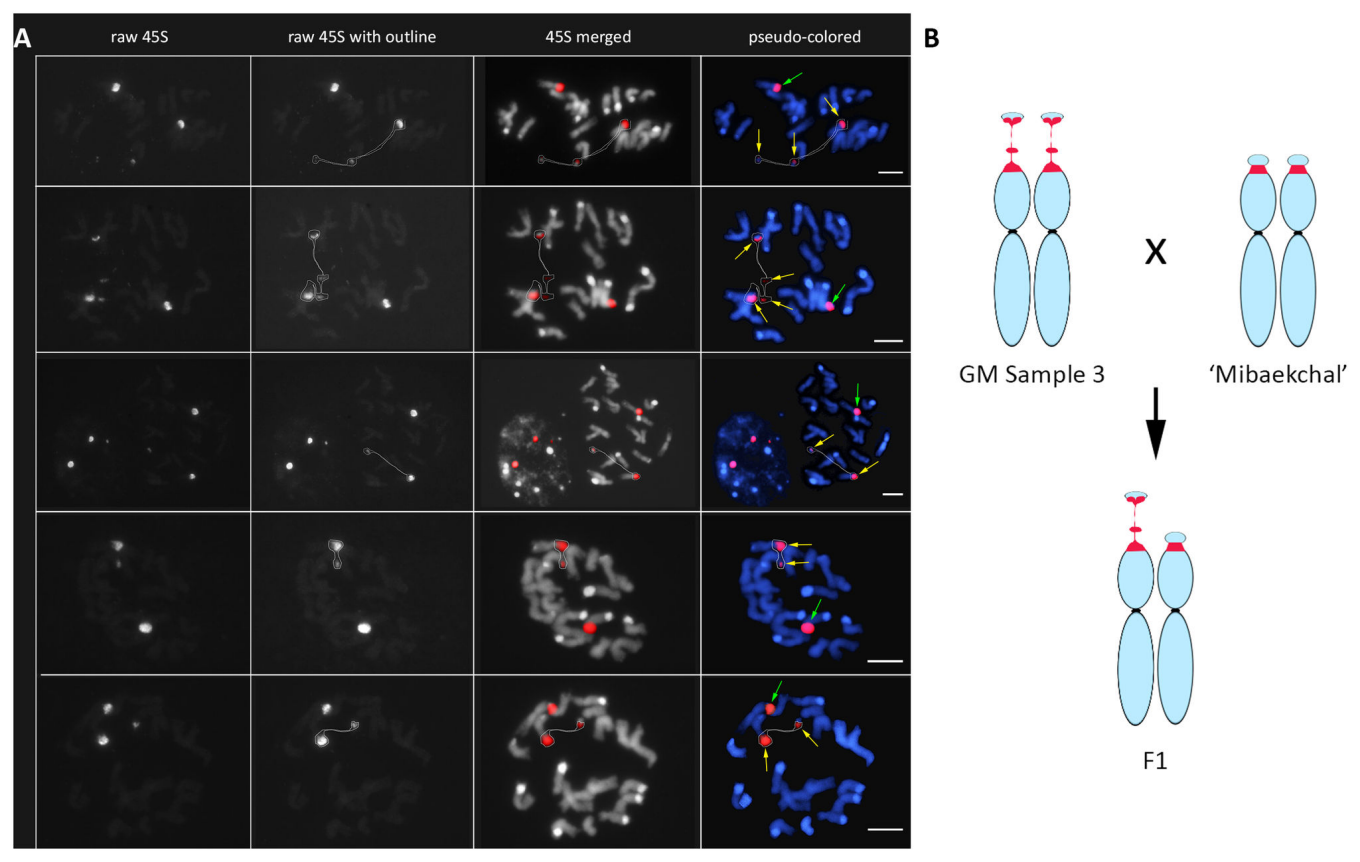
Genetic pattern of the fragile 45S rDNA locus. Panel A: FISH analysis of the 45S rDNA locus in F_1_ metaphase chromosome spreads show one satellited homologue with intact NOR (green arrows), and a fragmented NOR (yellow arrows) from its homologous locus. Bars = 5µm. B: A genetic diagram depicting the inheritance of NOR condensation pattern to the F_1_ progeny.

To further investigate whether the 45S rDNA fragility observed in GM sample 3 is heritable, we did a cross between GM Sample 3 and ‘Mibaekchal’. The F1 progeny showed a heterozygous 45S rDNA condensation pattern from each parent (Figure 7). One homologous locus showed an intact NOR signal at both interphase and metaphase, which corresponded to the pattern of ‘Mibaekchal’. Another homologue showed a fragmented pattern corresponding to the GM Sample 3. Consequently, three signals were observed frequently at interphase (97%) and metaphase (95%) of the F_1_ progeny (Figure 7, Figure S5, Figure S6 and Table 2). This indicates the heritability of the fragile 45S rDNA phenotype, and implies inheritance of its corresponding associated epigenetic mechanisms.

### Event-specific expression of 45S rDNA fragility

**Figure 8:**
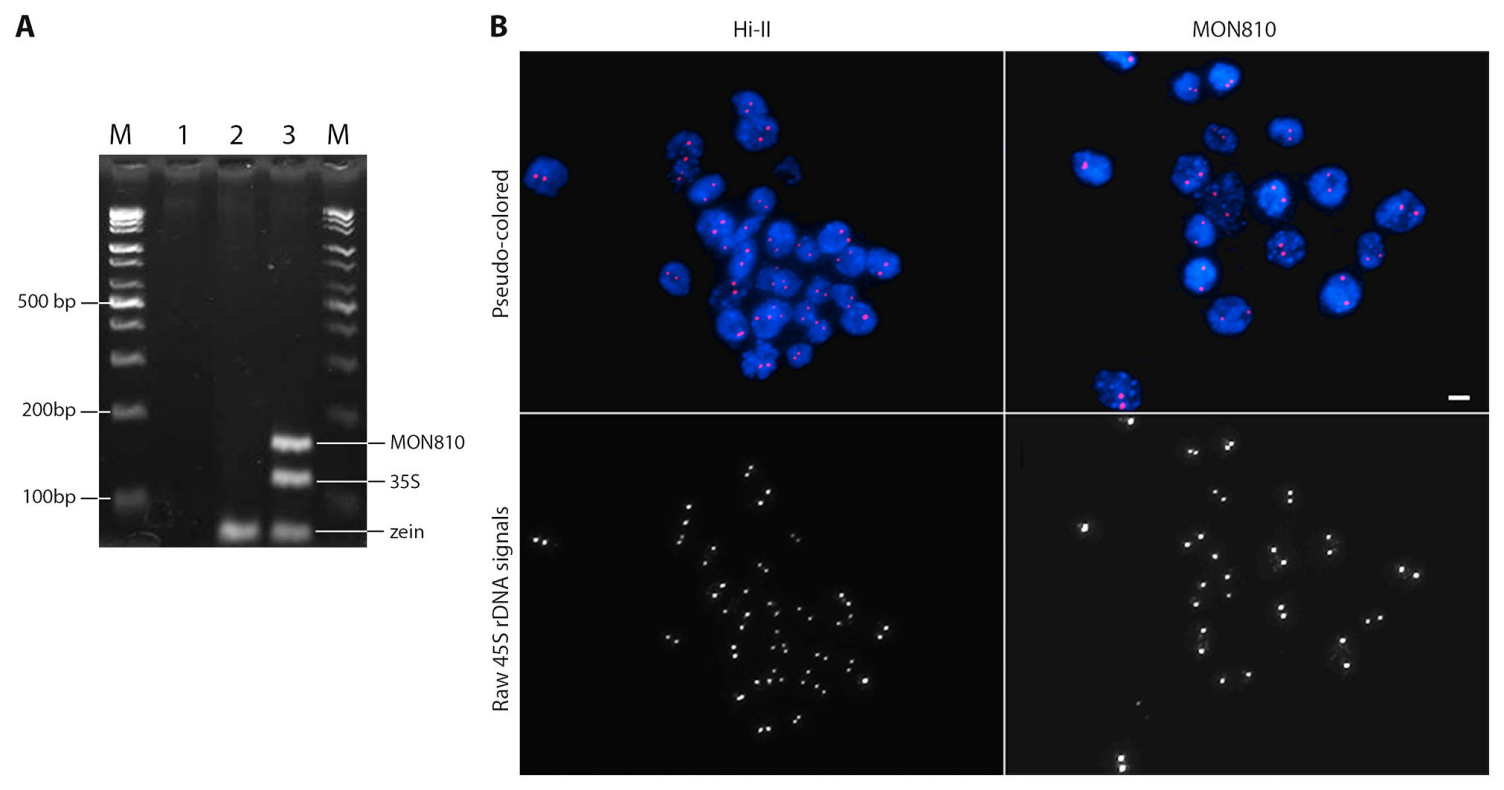
Comparative PCR and FISH analyses between Hi-II and MON810. Panel A: Multiplex PCR results confirmed the absence or presence of transgene in Hi-II and MON810, respectively. Lane N: negative control (no DNA template), 1: Hi-II, and 2: MON810. B: FISH analysis of the 45S rDNA cluster showed two intact signals in both Hi-II and MON810. Bar = 20µm.

To address the question of whether the 45S rDNA fragility observed in GM Sample 3 could exemplify other GM maize events, we investigated the 45S locus of GM maize event MON810 for any fragile pattern similar to that observed in GM Sample 3, and compared the results with its non-GM near isogenic line Hi-II. Among 903 and 497 nuclei observed in Hi-II and MON810, respectively, 896 (99%) and 489 (98%) showed two intact signals and no fragmentation (Figure 8, Table S3). This demonstrates that the fragility observed in GM Sample 3 could not exemplify other GM maize events, and suggests that fragility of the 45S rDNA locus could be event-specific.

## Discussion

The use of GM crops for food, feed, and processing has been the subject of intense debate since their initial introduction into the market [48]. Although many studies have concluded that GM crops were safe and substantially equivalent to their near isogenic lines [49], several investigations have revealed potential adverse side effects to the mammalian system by a diet containing GM products (e.g. [50,51]). Despite this dilemma, the global area used for cultivation of GM crops increased by a 100-fold from 1.7 million hectares in 1996 to 170 million in 2012 [5], indicating that both industrialized and developing countries have confidence in GM crops. Nevertheless, persistent concerns have led different countries to formulate varying guidelines for the use of GMOs [15].

In Korea, although GM crop cultivation has not yet been legalized [16,23], 86 GM crops including 44 GM maize events have already been approved for consumption [18]. Despite the ban on cultivation, the adventitious presence of LMOs in the environment is not impossible due to inadvertent release during shipping and handling. Several monitoring reports have confirmed the environmental presence of LMOs in the form of seeds and established plants in Incheon, Korea through multiplex PCR [16,19,23,52].

All events of GM maize imported to Korea contain the *35S* (or *e35S*) promoter, the *nos* terminator or both [16]; therefore, we used these segments to detect the presence of GM maize. Specifically, the absence of these GM-specific bands among field samples indicates that there has been no introgression of the transgenes into the sampled maize plants from different sites in Korea. However, the detection of viable GM kernels along transit roads near the importation ports indicates that transgene “leak” to the environment has occurred, which is of concern to environmentalists and legislators [47,53]. A few studies in Mexico, the origin and center of diversification of maize, have revealed introgression of transgenes into local landraces [54–56]. Although Korea is not an origin of maize diversity and no threatened wild relatives are present, preservation of local cultivars from transgene contamination will require monitoring with updated methodology since failure to detect the presence of transgenes does not necessarily indicate their absence [55].

Additionally, our cytogenetic observations of GM sample 3 revealed more chromosomal fragmentations at the 45S rDNA cluster than the non-GM sample, ‘Mibaekchal’. Interestingly, these fragmentations resemble the induced fragile 45S rDNA clusters reported by Huang et al. [33] for barley, maize, rice, ryegrass, and sorghum. These authors referred to these fragmentations as “chromosome lesions,” which were expressed after treatment of plant seedlings with inhibitors for transcription (actinomycin D) and replication (aphidicolin). Additionally, they showed that the non-treated control cells revealed normal number and condensation patterns of the 45S rDNA clusters (i.e. two loci in maize), while the cells induced for transcription and replication showed a “beads-on-a-string” fragmentation pattern. The induced instability of the 45S rDNA cluster was shown to be partly dependent on epigenetic modifications such as site-specific DNA hypomethylation and histone modifications [33]. Similarly, our data show that even with the absence of chemical induction, fragile 45S rDNA phenotype was homozygously expressed in GM Sample 3 (Figure 5, F–I), indicating that an endogenous mechanism, such as epigenetic alterations, could be responsible for its expression.

Furthermore, it has been reported that epigenetic mutation of an allele of the same gene (epiallele) could be actively repaired based on the pattern of the wild type epiallele [57], but in some context no restoration occurs, thereby resulting to stable epigenetic polymorphism between alleles [58], which could be passed on across generations [57,59–62]. Accordingly, the observation of a heterozygous 45S rDNA condensation patterns in the F_1_ generation not only demonstrated the heritability of the fragile 45S rDNA from GM Sample 3 and its subsequent expression in the F_1_ but also showed that no restoration of the fragile 45S rDNA epiallele to the wild type has taken place in the F_1_ genome. This implies that associated epigenetic changes or endogenous mechanisms responsible for its expression remained unchanged and uninfluenced by the wild type epiallele in the F_1_.

In the context of genetic engineering, it may be useful to note that transformation itself is known to be mutagenic [63–65], and during plant transformation, each transformation event receives a unique transgene integration pattern, and thus, a unique accompanying genomic rearrangement [38,40,66–69]. Thus, it is logical to treat each event individually and uniquely. Moreover, numerous non-GM inbred lines and varieties commonly used in maize breeding, both from literature [33,70,71] and unpublished data from our lab (‘Paksachal’, Figure S7), have shown intact 45S rDNA locus. This implies the likely event-specificity of the expression of fragile 45S rDNA, while not denying the possibility that this fragility could be observed only in this particular event while absent in other GM maize events. This may explain why we did not observe a fragile 45S rDNA phenotype in MON810 similar to what have been observed in GM sample 3. Putting these together, since GM Sample 3 contained the NK603 and MON810 transgenes, it is likely that fragile 45S rDNA was caused by the NK603 integration event. Linkage experiments to evaluate the correlation between NK603 and 45S rDNA fragility is logically the next step in order to confirm this hypothesis. Meanwhile, a recent report about the higher rate of kidney and liver-related diseases and higher occurrence of tumor in rats fed with NK603-enriched rat chows compared with the control groups [50] has made this subject an even more interesting topic for further research.

Changes in the epigenetic machinery are also associated with changes in genome function, which could be produced by transposon activation [72], polyploidization [73], or genetic modification [39]. Physical association of abundant transposable elements around the 45S rDNA cluster has been linked to increase in fragility of this locus [74]. This supports the possible pathway of fragile 45S rDNA expression which starts from genomic rearrangement that leads to transposable element activation [72], to insertion of transposable elements within or around the 45S rDNA locus [75–79], and ultimately the expression of fragility at this locus [74]. Another possible pathway would be from the epigenetic alterations caused by genome rearrangement [39] that lead to defective transcription and/or replication machinery [33], and finally to its expression.

In a study of the significance of the 45S rDNA cluster in the *Drosophila* genome, Paredes et al. [32] demonstrated that hundreds to thousands of genes could be differentially expressed depending on the copy number of the rDNA genes, and that some genes situated across the genome are responsive to changes in the rDNA condition. Accordingly, disruption of the rDNA cluster could have more profound effects than traditionally expected [32].

In addition, genetic modification could also affect portions of the proteomes such as novel fusion proteins [39,40]. Although proteomic profiling may be used to screen the safety of GM products, it has not been used routinely and still cannot accurately identify novel proteins beyond those predicted to be present in a plant proteome [39].

Given the multifaceted effects of genetic modification, from DNA sequence to protein levels [63,64], our yet limited understanding of genomic and epigenomic interactions, and limited available cytogenetic data related to genetically engineered maize, further investigations of relationships between crop genetic engineering and regulatory molecules controlling the mechanism for the expression of 45S rDNA fragile phenotype may prove necessary and interesting.

Here, we report the presence of GM maize inadvertently dispersed into the environment by qualitative multiplex PCR. Based on this finding, we suggest that regular monitoring be conducted to control the unwanted flow of transgenes into local Korean maize cultivars. Additionally, the expression of a fragile 45S rDNA phenotype in GM Sample 3 could imply underlying epigenetic alterations linked to genetic modification. Based on several reports linking genetically modified foods [50], chromosomal fragile sites in general [30], and abnormality in the rDNA transcription machinery [80] to cancer development in mammals, careful multifaceted investigations of the influence of plant genetic modification on the crop genome and proteome integrity and ultimately the human diet and health are warranted.

## Supporting Information

Figure S1 **FISH analysis of the 45S rDNA cluster in the interphase nuclei of GM sample 3.** The 45S rDNA hybridization patterns revealed three to six fragments. Expected loci are shown in the first row. Yellow arrows emphasize the 45S rDNA signals. Bar = 5µm.
(TIF)

Figure S2 **The 45S rDNA hybridization patterns on the GM sample metaphase chromosomes showing the “bead-on-a-string” pattern of fragmentation.** Raw signals, before and after outlining of the “string” chromatin, are shown in the first and second column, respectively, and 45S rDNA signals overlaid on raw DAPI images and pseudo-colored images are shown in the third and fourth columns. Rows A-D show different metaphase spreads with the three fragmentation pattern of the rDNA cluster. Bar = 5µm.
(TIF)

Figure S3 **The “beads-on-a-string” pattern of the 45S rDNA signal on GM sample 3 metaphase spread.** Note the small ‘bead’ (yellow arrows) connected by two “strings” (green arrows) in panel A, forming a ring-like structure. Panel B: 45S rDNA signal overlaid on the raw DAPI image, C: Pseudo-colored image reduces the beads-on-a-string signal. Satellite chromosomes are indicated by the number 6, and asterisks represent the homologue bearing a large knob at the distal portion of the long arm. White arrowhead indicates the NOR site with lost satellite arm. Bar = 5µm.
(TIF)

Figure S4 Metaphase spreads of ‘Mibaekchal’ (**A**) and GM sample 3 (**B**) and their karyotypes (**C**). The 5S (green) and 45S (red) signals are shown. White arrowheads indicate the NOR site with lost satellite arm in GM sample 3. Bars = 5µm.
(TIF)

Figure S5 **Comparison of the genotype, phenotype, and 45S rDNA fragility between the parents and F_1_.** GM sample 3 had yellow endosperm and contained NK603 (~400bp and ~800 bp) and MON810 bands, which were both passed on to the F_1_. ‘Mibaekchal’ had white endosperm and did not contain any transgene-specific band. Most of the nuclei observed in GM Sample 3 had homozygously fragmented 45S rDNA (arrows) compared with intact sites in ‘Mibaekchal’. Heterozygous fragility in the F_1_ indicates the inheritance and expression of fragile 45S rDNA phenotype and its underlying mechanisms.
(TIF)

Figure S6 **FISH analysis of the 45S rDNA cluster in the interphase nuclei of the F_1_ plants.** The 45S rDNA hybridization patterns revealed three fragments. Arrows emphasize the 45S rDNA signals. Bar = 5µm.
(TIF)

Figure S7 **FISH analysis of the 45S rDNA cluster in the interphase (**A**) and metaphase (**B**) of Korean maize cultivar ‘Paksachal’.** No fragility of the 45S rDNA locus was observed. Green and red dots represent 5S and 45S rDNA loci, respectively. Bar = 5µm.
(TIF)

Table S1 List of primers used in this study.
(DOCX)

Table S2 2SD-mPCR cycling conditions used for GM detection.
(DOCX)

Table S3 **Comparison of 45S rDNA cluster fragility on interphase nuclei between MON810 and its non-GM isogenic line Hi-II.**
(DOCX)
