# Supplementary figures and images for: Randomly Detected Genetically Modified (GM) Maize (Zea mays L.) near a Transport Route Revealed a Fragile 45S rDNA Phenotype
Source: PLoS One. 2013 Sep 9;8(9):e74060. doi: 10.1371/journal.pone.0074060 (PMC3767626; doi:10.1371/journal.pone.0074060)

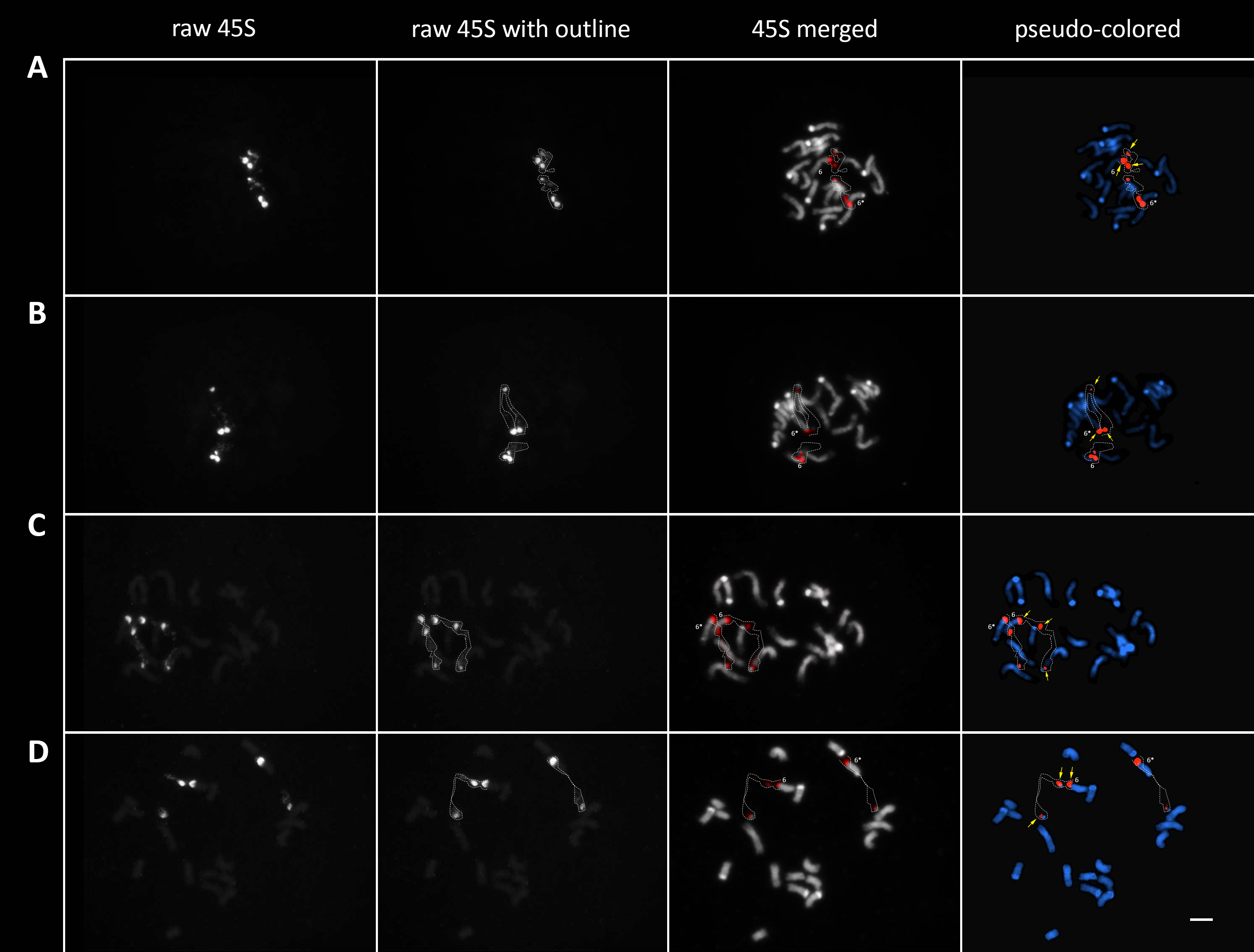

Supplement: Figure S2 — The 45S rDNA hybridization patterns on the GM sample metaphase chromosomes showing the “bead-on-a-string” pattern of fragmentation. Raw signals, before and after outlining of the “string” chromatin, are shown in the first and second column, respectively, and 45S rDNA signals overlaid on raw DAPI images and pseudo-colored images are shown in the third and fourth columns. Rows A-D show different metaphase spreads with the three fragmentation pattern of the rDNA cluster. Bar = 5µm. (TIF) [file pone.0074060.s002.tif]

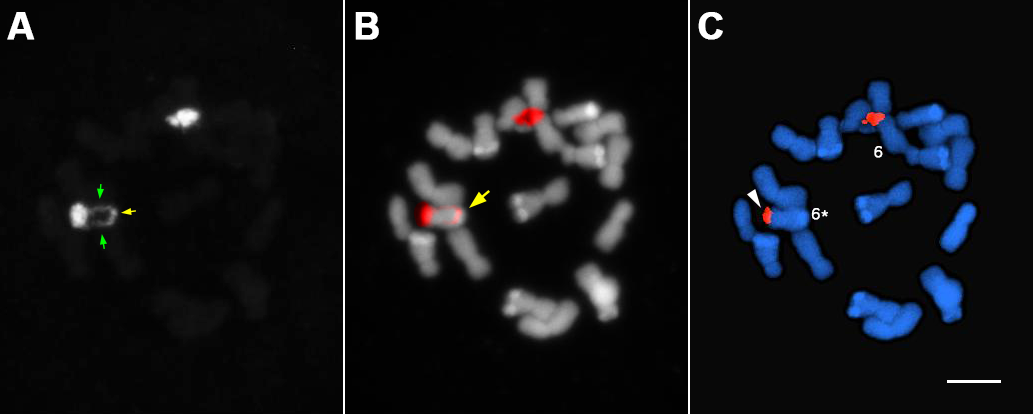

Supplement: Figure S3 — The “beads-on-a-string” pattern of the 45S rDNA signal on GM sample 3 metaphase spread. Note the small ‘bead’ (yellow arrows) connected by two “strings” (green arrows) in panel A, forming a ring-like structure. Panel B: 45S rDNA signal overlaid on the raw DAPI image, C: Pseudo-colored image reduces the beads-on-a-string signal. Satellite chromosomes are indicated by the number 6, and asterisks represent the homologue bearing a large knob at the distal portion of the long arm. White arrowhead indicates the NOR site with lost satellite arm. Bar = 5µm. (TIF) [file pone.0074060.s003.tif]

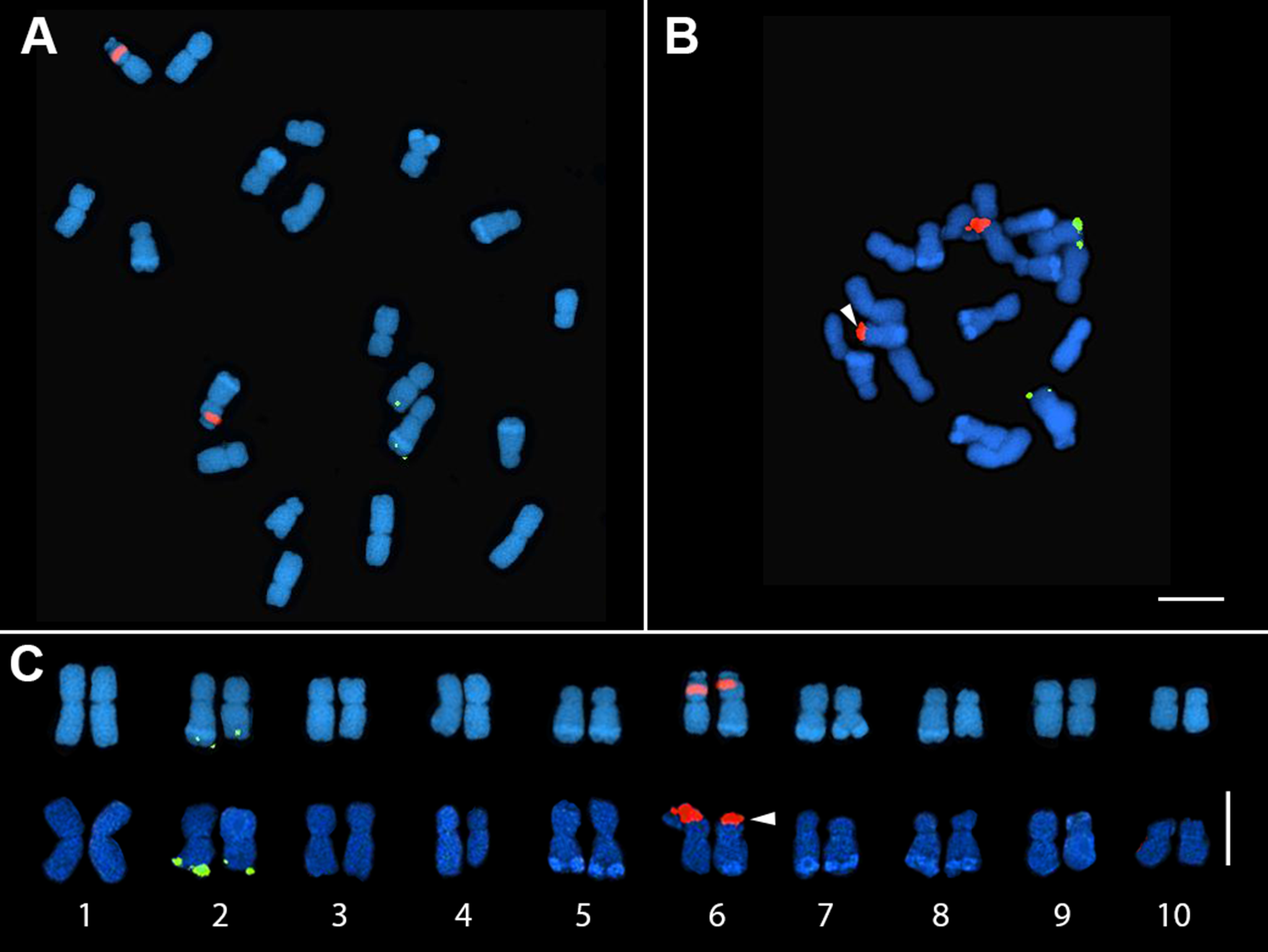

Supplement: Figure S4 — Metaphase spreads of ‘Mibaekchal’ (A) and GM sample 3 (B) and their karyotypes (C). The 5S (green) and 45S (red) signals are shown. White arrowheads indicate the NOR site with lost satellite arm in GM sample 3. Bars = 5µm. (TIF) [file pone.0074060.s004.tif]

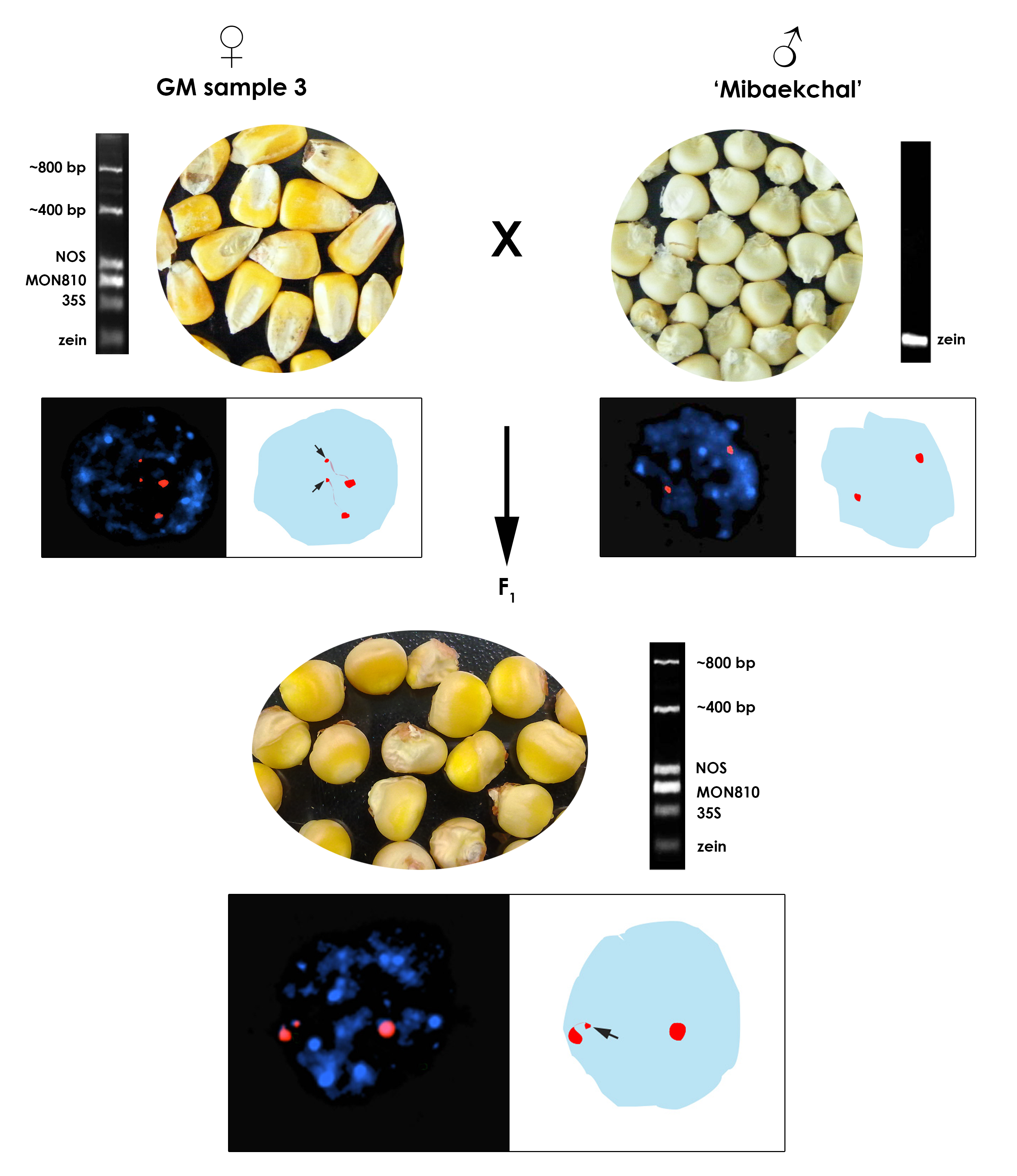

Supplement: Figure S5 — Comparison of the genotype, phenotype, and 45S rDNA fragility between the parents and F1. GM sample 3 had yellow endosperm and contained NK603 (~400bp and ~800 bp) and MON810 bands, which were both passed on to the F1. ‘Mibaekchal’ had white endosperm and did not contain any transgene-specific band. Most of the nuclei observed in GM Sample 3 had homozygously fragmented 45S rDNA (arrows) compared with intact sites in ‘Mibaekchal’. Heterozygous fragility in the F1 indicates the inheritance and expression of fragile 45S rDNA phenotype and its underlying mechanisms. (TIF) [file pone.0074060.s005.tif]

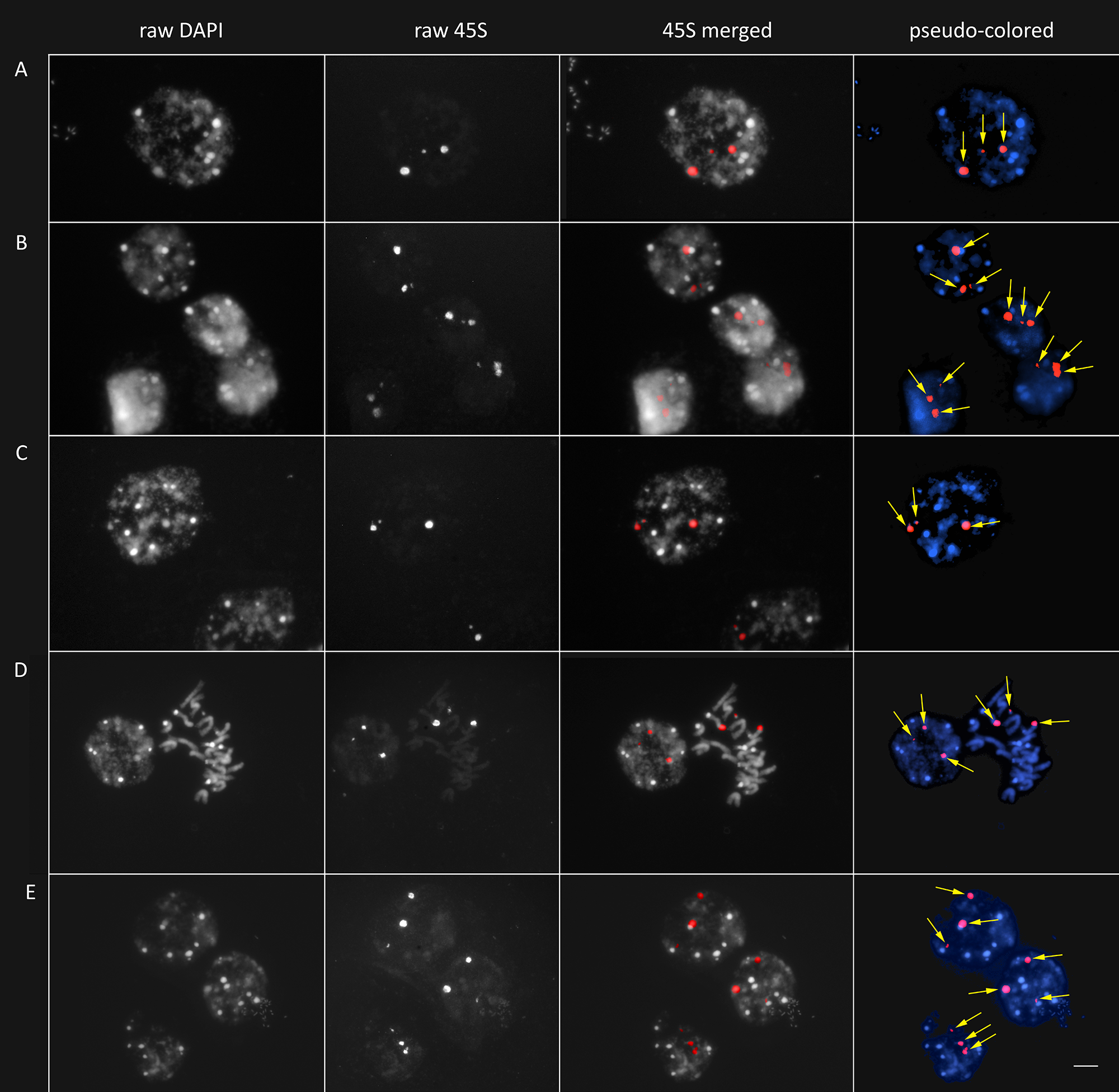

Supplement: Figure S6 — FISH analysis of the 45S rDNA cluster in the interphase nuclei of the F1 plants. The 45S rDNA hybridization patterns revealed three fragments. Arrows emphasize the 45S rDNA signals. Bar = 5µm. (TIF) [file pone.0074060.s006.tif]

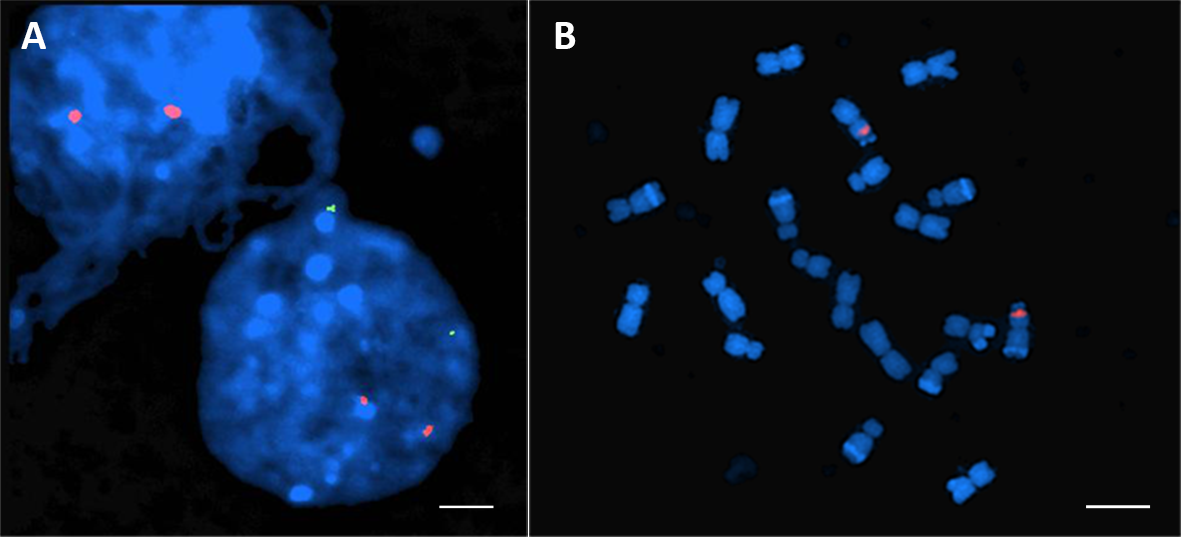

Supplement: Figure S7 — FISH analysis of the 45S rDNA cluster in the interphase (A) and metaphase (B) of Korean maize cultivar ‘Paksachal’. No fragility of the 45S rDNA locus was observed. Green and red dots represent 5S and 45S rDNA loci, respectively. Bar = 5µm. (TIF) [file pone.0074060.s007.tif]
