# Supplementary material for: Randomly Detected Genetically Modified (GM) Maize (Zea mays L.) near a Transport Route Revealed a Fragile 45S rDNA Phenotype
Source: PLoS One. 2013 Sep 9;8(9):e74060. doi: 10.1371/journal.pone.0074060 (PMC3767626; doi:10.1371/journal.pone.0074060)
Supplement: Table S1 — List of primers used in this study. (DOCX) [file pone.0074060.s008.docx]

Table S1. List of primers used in this study.

| **Target** | **Primer name** | **Sequence** | **Expected product size (bp)** | **Reference** |
| --- | --- | --- | --- | --- |
| MON810 | MON810F (sense) | 5’ CAC CAC AGC CAC CAC TTC T 3’ | 150 | [[27](#_ENREF_27)] |
|  | MON810R (antisense) | 5’ AGG AAA AGC TAT TGT AAA GCC AAA 3’ |  |  |
| MON863 | MON863F (sense) | 5’ GTA ATC GGC TAA TCG CCA AC 3’ | 224 | [[27](#_ENREF_27)] |
|  | MON863R (antisense) | 5’ GCC AGT TCA TTG CGA GTA CA 3’ |  |  |
| NK603 | NK603F (sense) | 5’ GAGTTTCCTTTTTGTTGCTCTC 3' | 444 | [[20](#_ENREF_20)] |
|  | NK603R (antisense) | 5’ GCTGCTTGCACCGTGAAG 3’ |  |  |
| *35S* | 35SF (sense) | 5’ GAC AGT GGT CCC AAA GAT GGA C 3’ | 115 |  |
|  | 35SR (antisense) | 5’ CCT TAC GTC AGT GGA GAT ATC 3’ |  |  |
| *nos* | NOSF (sense) | 5’ CTG TTG CCG GTC TTG CGA TG 3’ | 185 |  |
|  | NOSR (antisense) | 5’ GCG CGA TAA TTT ATC CTA GTT TG 3’ |  |  |
| *zein* | zeinF (sense) | 5’ GCT TGC GGA GCT TGA TGG CGT 3’ | 72 | [[27](#_ENREF_27)] |
|  | zeinR (antisense) | 5’ GGC ATC GTC TGA AGC GGT AAG G 3’ |  |  |
