# Supplementary material for: Randomly Detected Genetically Modified (GM) Maize (Zea mays L.) near a Transport Route Revealed a Fragile 45S rDNA Phenotype
Source: PLoS One. 2013 Sep 9;8(9):e74060. doi: 10.1371/journal.pone.0074060 (PMC3767626; doi:10.1371/journal.pone.0074060)
Supplement: Table S2 — 2SD-mPCR cycling conditions used for GM detection. (DOCX) [file pone.0074060.s009.docx]

**Table S2.** 2SD-mPCR cycling conditions used for GM detection.

| **Step** | **Process** | **Duration** | **Temperature** | **Cycles** |
| --- | --- | --- | --- | --- |
| Initial denaturation |  | 15 min. | 95°C |  |
| Step 1 | Denaturation | 30 s | 94°C | x15 |
|  | Annealing | 90s | 62°C |  |
|  | Extension | 90s | 72°C |  |
| Step 2 | Denaturation | 30 s | 94°C | x20 |
|  | Annealing | 90s | 57°C |  |
|  | Extension | 90s | 72°C |  |
| Final extension |  | 10 min. | 72°C |  |
