# Supplementary material for: Randomly Detected Genetically Modified (GM) Maize (Zea mays L.) near a Transport Route Revealed a Fragile 45S rDNA Phenotype
Source: PLoS One. 2013 Sep 9;8(9):e74060. doi: 10.1371/journal.pone.0074060 (PMC3767626; doi:10.1371/journal.pone.0074060)
Supplement: Table S3 — Comparison of 45S rDNA cluster fragility on interphase nuclei between MON810 and its non-GM isogenic line Hi-II. (DOCX) [file pone.0074060.s010.docx]

**Table S3.** Comparison of 45S rDNA cluster fragility on interphase nuclei between MON810 and its non-GM isogenic line Hi-II.

| **Sample** | **Intact^a^** | **Fragmented** | | **Total** |
| --- | --- | --- | --- | --- |
| Hi-II | 896 (99.2)^b^ | 7 (0.8) | | 903 |
| MON810 | 489 (98.4) | 8 (1.6) | | 497 |
| Total |  |  |  | 1400 |

^a^ Two distinct signals

^b^ Values inside the parentheses are percentages
